# Oxidative Damage as a Fundament of Systemic Toxicities Induced by Cisplatin—The Crucial Limitation or Potential Therapeutic Target?

**DOI:** 10.3390/ijms241914574

**Published:** 2023-09-26

**Authors:** Jelena S. Katanić Stanković, Dragica Selaković, Gvozden Rosić

**Affiliations:** 1 Department of Science, Institute for Information Technologies Kragujevac, University of Kragujevac, Jovana Cvijića bb, 34000 Kragujevac, Serbia; 2 Department of Physiology, Faculty of Medical Sciences, University of Kragujevac, Svetozara Markovića 69, 34000 Kragujevac, Serbia; dragica984@gmail.com

**Keywords:** cisplatin, oxidative stress, toxicity, animal models, perspectives

## Abstract

Cisplatin, an inorganic complex of platinum, is a chemotherapeutic drug that has been used for 45 years. Despite the progress of pharmaceutical sciences and medicine and the successful application of other platinum complexes for the same purpose, cisplatin is still the therapy of choice in many cancers. Treatment for testicular, ovarian, head and neck, urothelial, cervical, esophageal, breast, and pulmonary malignancies is still unthinkable without the use of this drug. However, cisplatin is also known for many side effects, of which the most pronounced are nephrotoxicity leading to acute renal failure, neurotoxicity, and ototoxicity. Mechanistic studies have proven that one of the conditions that plays a major role in the development of cisplatin-induced toxicities is oxidative stress. Knowing the fact that numerous antioxidants can be used to reduce oxidative stress, thereby reducing tissue lesions, organ failure, and apoptosis at the cellular level, many studies have defined antioxidants as a priority for investigation as a cotreatment. To investigate the mechanism of antioxidant action in vivo, many animal models have been employed. In the last few years, studies have mostly used rodents and zebrafish models. In this article, some of the most recent investigations that used animal models are listed, and the advantages and disadvantages of such experimental studies are pointed out.

## 1. Introduction

### 1.1. Definition of Oxidative Damage

To understand the concept of oxidative damage, several factors must be understood. Free radicals are notably unstable and exhibit considerable nonselective reactivity with other molecular species [1]. The concept of free radicals was initially introduced by Moses Gomberg in 1900 when he researched trivalent carbon [2]. It was widely believed that these highly reactive and short-lived entities were absent from biological systems but their role as free radicals in biological processes and involvement in pathological processes and aging phenomena is substantial [3].

Our understanding of free radicals has since expanded considerably; they are now acknowledged as active participants in a multitude of life processes within an array of organisms. Furthermore, their function should not solely be perceived as deleterious but also as integral components of numerous normal physiological functions. There are both endogenous and exogenous sources for the generation of free radicals. Within living organisms (endogenously), free radicals are produced as a natural byproduct of regular metabolic processes within mitochondria. They can also arise due to xanthine oxidase activity, peroxisome function, ischemic events, phagocytosis, inflammatory reactions, and the arachidonic acid pathway. On the other hand, various external factors influence free radical production; these include medications, environmental contaminants such as pollution or pesticides, tobacco smoke exposure, physical exertion diverse forms of radiation like ultraviolet or ionizing radiation, industrial solvent usage, and ozone concentrations [1,4]. By clarifying our understanding of these highly reactive molecular species’ roles and sources, we can now perceive free radicals from a more holistic perspective instead of purely as detrimental agents. In biological systems, free radicals have three elements in their structure: oxygen (O), nitrogen (N), and sulfur (S); thus, there can be distinguished reactive oxygen species (ROS), reactive nitrogen species (RNS), and reactive sulfur species (RSS) [1,5]. What is a great irony is that these elements are essential for life, and they are generated in normal aerobic metabolism, but due to the formation of free radicals, they also may have harmful effects on the organism. In addition to free radicals (superoxide anion radical, O_2_^−·^; hydroxyl radical, HO^·^; nitrogen oxide radical, NO^·^; and alkyloxy radical, RO^·^), reactive species also include extremely reactive nonradical forms, such as hydrogen peroxide and organic peroxides, singlet oxygen, ozone, peroxynitrites, thiols, and thiosulfonates [5,6].

The negative side of free radicals is certainly their great reactivity towards all classes of primary biomolecules (proteins, carbohydrates, lipids, and nucleic acids) [7,8]. They can react with other stable molecules in numerous ways and different types of reaction mechanisms [1]. The reason for the manifestation of the harmful effects of free radicals lies in the origin and development of oxidative stress, a process in the body when the redox homeostasis in cells is disturbed, i.e., the state when the production of these reactive species overcomes the body’s defense mechanisms [5]. In general, the body has a balanced system of free radical production and activities of antioxidants, compounds that act against free radicals, whereby reactive species exhibit their beneficial properties, while the negative consequences are mostly reduced [4]. Therefore, if the established balance is disturbed, either by the hyperproduction of free radicals (especially ROS) or a reduced amount and activity of enzymatic and nonenzymatic antioxidants, symptoms of oxidative stress appear in cells [1,9]. This complex process has a deleterious influence on the organism that depends upon several factors, including the specific category of the oxidizing agent, the precise location and intensity of its generation, the composition, and functions of a multitude of antioxidant substances present, as well as the efficiency and efficacy of cellular repair mechanisms in response to oxidative stress [9]. The mitochondrion is recognized as the principal cellular organelle responsible for the production of ROS. Within the mitochondria, adenosine triphosphate (ATP) is generated via a series of oxidative phosphorylation processes. The deviation in this process results in the formation of O_2_^∙^ or H_2_O_2_, which can subsequently be converted into other ROS. It is important to note that additional sources of ROS production include the reactions involving cytochrome P-450 enzymes, NAD(P)H oxidases, peroxisomal oxidases, and xanthine oxidase. These alternative pathways further contribute to the complex landscape of ROS generation within cells [3,9].

During instances of pathological conditions, an overproduction of free radicals is observed, which can be attributed to the presence of prooxidant compounds and various other risk factors such as tobacco consumption, excessive physical activity, elevated stress levels, and other contributing elements. This phenomenon leads to the development of the well-studied oxidative stress, having significant implications for cellular and molecular processes. From this state, many serious physiological ailments can be developed because of tissue damage, ischemia/reperfusion, and hypoxia-induced oxidative stress, e.g., heart failure, stroke, angina, hypertension, Alzheimer, Parkinson, Wilson’s disease, rheumatoid arthritis, multiple sclerosis, infertility, diabetes, cataracts, asthma, allergies, etc. [1,3]. The disturbance in redox equilibrium affects cell homeostasis, signal transduction, gene expression, receptor activation, and pathogen recognition. Moreover, oxidative stress is one of the most prominent underlying factors for cancer development. The ROS synthesis in mitochondria initiates cellular redox signaling processes that correlate to proliferative cellular responses. The activation of transcription factors contributes to the promotion of tumorigenesis, and mitochondrial DNA may experience damage caused by ROS, leading to genetic mutations that have been identified as regulators of the tumorigenic phenotype [10].

### 1.2. Cisplatin and Its Mechanism of Action

**Figure 1:**
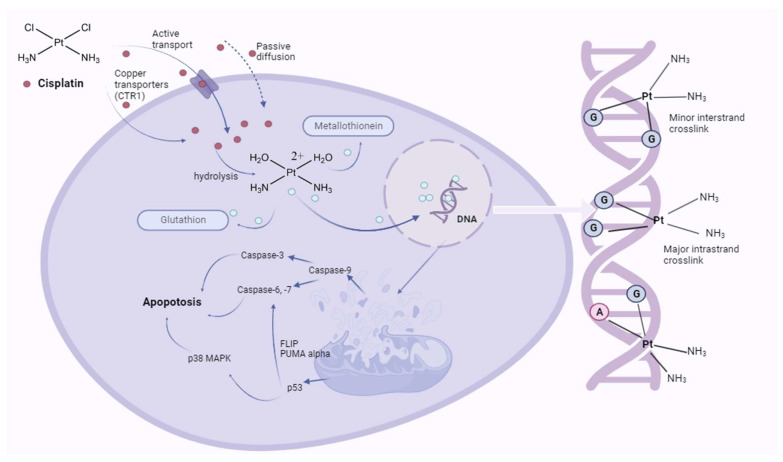
Cisplatin action within the cell. Created with BioRender.com (accessed on 11 September 2023). Red dots, cisplatin; blue dots, hydrolyzed cisplatin.

Cisplatin (cis-diamminedichloridoplatinum (II)) is an inorganic complex that exhibits a square planar molecular geometry. In this structure (cis-[Pt(NH_3_)_2_Cl_2_]), a central platinum (II) ion is coordinated by two chlorine atoms and two ammonia ligands situated in the cis-configuration. Since its approval by the United States Food and Drug Administration (FDA) in 1978, cisplatin has been employed extensively as a chemotherapeutic agent for various malignancies. Presently, it is part of the World Health Organization’s list of essential medicines [11,12,13]. The underlying mode of action for cisplatin’s antineoplastic properties involves its reaction and binding to deoxyribonucleic acid (DNA) within cells, which causes irreversible apoptosis (Figure 1). It has been hypothesized that the primary means of cellular uptake for cisplatin occurs passively via diffusion; however, the use of the plasma membrane copper transporter 1 (Ctr1p) for active transport may also contribute. Upon entering the cell and losing two chloride ions, it transforms into a reactive complex that readily bonds with DNA. This interaction results in both intra-strand and inter-strand DNA crosslinks resistant to DNA repair mechanisms and localized denaturation, thereby impeding further DNA replication and transcription processes, ultimately exerting its cytotoxic effect [14,15,16]. Cisplatin, a highly efficacious antineoplastic agent, has been extensively employed in the treatment of a diverse range of neoplasms, with a primary focus on testicular, ovarian, head and neck, urothelial, cervical, esophageal, breast, and pulmonary malignancies [15].

## 2. Oxidative Damage in Systemic Toxicity Induced by Cisplatin

**Figure 2:**
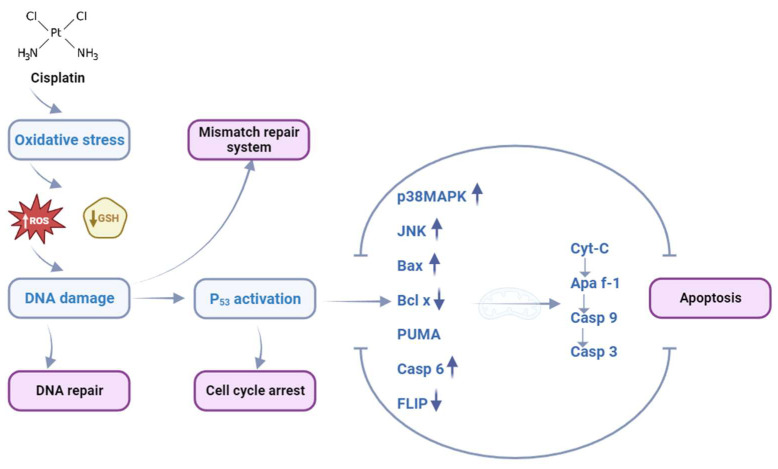
The basic mechanism of cisplatin action within the cell, modified from [11,17]. Created with BioRender.com (accessed on 11 September 2023).

The preferential bonding sites for cisplatin within DNA include the N-7 and O-6 atoms of neighboring guanine (G) molecules, as well as the N-7 and N-1 atoms of adenine (A) or, less frequently, the N-3 atom of cytosine (C). Notably, the main product responsible for cisplatin’s anticancer activity is the formation of 1,2-guaninedeoxynucleotide (GpG) adducts. In these adducts, platinum atoms are intrachain-coordinated to the N(7) atoms of guanine moieties from adjacent DNA chains. The formation of DNA adducts precedes the process of DNA damage identification by various proteins that effectively convey DNA damage signals to downstream signaling pathways, including the p53, mitogen-activated protein kinase (MAPK), and p73 pathways, ultimately resulting in apoptosis. Although there are available DNA repair mechanisms, the cells are, in most cases, susceptible to apoptotic or nonapoptotic cell death. The toxicity occurs via a cascade mechanism that begins with the development of oxidative stress due to the modulation of calcium signaling. The next step is mitochondrial dysfunction, where the activation of executioner caspases occurs followed by the development of apoptosis [11,17]. Moreover, cisplatin instigates apoptosis and impedes the proliferation of stem cells by elevating the expression levels of proapoptotic genes, concurrently attenuating the expression of one of the key antiapoptotic genes, Bcl-2. Cisplatin forms a bond with mitochondrial DNA (mtDNA), triggering irreversible damage that consequently leads to the obstructed replication and transcription of mtDNA, culminating in mitochondrial dysfunction and cellular demise. The impairment of mtDNA and ensuing mitochondrial dysfunction give rise to the generation of unbound reactive oxygen species (ROS) and trigger oxidative stress-mediated reactions [18,19,20,21,22] (Figure 2).

Therefore, while cisplatin plays a crucial role in cancer treatment, its administration is associated with myriad adverse effects, resulting in complications such as vomiting, gastrointestinal disorders, and toxic manifestations influencing multiple organs and systems. In the context of cisplatin therapy, nephrotoxicity, neurotoxicity, and ototoxicity are the most frequently observed toxicities, while instances of myelosuppression and hepatotoxicity may also occur [21,23,24,25]. The aforementioned toxic consequences of cisplatin stem from the compound’s affinity toward sulfur-containing molecules (e.g., glutathione), wherein thiol groups coordinate with the Pt (II) ion and subsequently impede cisplatin–DNA interactions. The resulting complexes exhibit high reactivity and are primarily accountable for cisplatin’s side effects [19,20]. Besides cisplatin binding to various cytoplasmic molecules, other proposed mechanisms underlying cisplatin-induced toxicity involve the elevated generation of ROS and inhibition of antioxidant enzymes, such as superoxide dismutase, glutathione peroxidase, and glutathione-S-transferase. Evidently, free radical formation, oxidative stress, inflammation instigated by free radical action, and the disruption of mitochondrial function contribute significantly to the manifestation of cisplatin’s toxic effects [26,27,28].

### 2.1. Role of Cisplatin-Induced Oxidative Stress in Tissue Injury

The role of cisplatin as a chemotherapeutic agent is extremely important. This can be seen, first of all, from the fact that, after over 40 years of its use and the large number of side effects it causes, cisplatin therapy is still the therapy of choice in a large number of malignant conditions. However, it is necessary to refer to the very serious side effects and organ toxicities that this drug causes. Namely, cisplatin can cause toxicities of different levels, from mild to highly severe conditions. The ones that are mentioned most often and that represent the biggest problem due to the seriousness of the conditions they cause are nephrotoxicity and peripheral neurotoxicity. Nephrotoxicity is recorded in 20–41% of patients and is mostly present in adults, and a similar situation is also related to peripheral neuropathy, which is present in adults in as many as 86% of cases. The category of younger patients and children is most often affected by the development of ototoxicity (it occurs in more than 50% of children receiving cisplatin therapy) [24].

There are *two aspects* of the generation of cisplatin toxicities. The first one arises from the cisplatin primary action, binding to the DNA, and the second aspect is directed towards the development of oxidative stress via the formation of free radicals and disruption of the inner defense mechanisms [16].

In addition to renal, peripheral nervous, and auditory systems, cisplatin therapy may also impact organs such as the hepatic system, cardiovascular system, and gastrointestinal tract. Myelosuppression, pronounced nausea, and emetic responses are potential side effects of this treatment as well. The problems that cisplatin causes in the gastrointestinal tract are different, and the most common occur very often, primarily related to nausea and vomiting. As a consequence, diarrhea, weight loss, and, in some patients, even anorexia can develop. Cisplatin may cause various hematological disorders, such as thrombosis, thrombocytopenia, neutropenia, leukopenia, and anemia, but also myelosuppression. From a clinical perspective, cisplatin may effectively treat specific tumors; however, it can also induce long-term toxicological consequences, causing secondary malignancies, particularly in cured testicular cancer patients [29].

When the focus is on the most common side effect of cisplatin therapy, *nephrotoxicity*, it should be noted that the kidney tissue retains the largest amount of cisplatin that enters the body—in some cases, even five times higher amount than serum. During the excretion of cisplatin through the kidneys, they accumulate extremely high concentrations of this drug, primarily in the proximal tubule epithelial cells, where it can enter via copper transporter Ctr1 and the organic cation transporter 2 [16]. Numerous studies have demonstrated that cisplatin induces damage to an array of renal components, encompassing the vasculature, glomerular apparatus, and, most prevalently, the renal tubules [30]. Depending on the duration and concentration of the treatment, cisplatin can cause necrosis or apoptosis of renal cells, leading to acute kidney injury and nephrotoxicity. The apoptosis pathway is triggered by ROS production and accumulation [24]. According to Hosohata [31], there are several ways of unfolding cisplatin-induced oxidative stress in kidney cells. The first path of accumulation of ROS in renal tissue is via the highly reactive cisplatin form. It has a high affinity towards the thiol functional group of compounds, particularly glutathione (GSH), which is known as one of the most important compounds in antioxidant defense. After cisplatin enters into a reaction with GSH, the breakdown of GSH molecules occurs, reducing its concentration and, therefore, decreasing the endogenous antioxidant activity of the organism. Because of this, there is an increase in the amount of ROS in the cells and the occurrence of oxidative stress. In addition, tubular cell death can occur due to the activation of many signaling pathways (MAPK, P53, and P21). All this can lead to the triggering of an inflammatory response, which additionally contributes to the accumulation of ROS in tissues and the development of fibrosis. Another factor that influences the high amounts of ROS in renal cells can be cisplatin-induced mitochondrial dysfunction. Finally, the increase in the amount of ROS and the occurrence of oxidative stress in the microsomes of kidney tissue can also occur due to the effect of cisplatin on the cytochrome P450 system [31].

*Neurotoxicity* is considered the second-most serious side effect of cisplatin treatment after nephrotoxicity. It is characterized by the primary sensory neuropathy originating from cisplatin-induced damage of the dorsal root ganglia of the spinal cord [24]. The first signs of development of a peripheral sensory neuropathy condition are aberrant sensory symptoms (pain, burning sensation, and decreased sensitivity) with a symmetric distribution in the extremities, specifically affecting the hands and feet, with potential extension to the elbows and knees. These symptoms can last long after the cisplatin therapy is over; thus, cisplatin-induced neuropathy can severely affect the quality of patients’ lives, causing damage even after treatment [16,24,25]. One of the proposed mechanisms of cisplatin-induced neuropathy development postulates that oxidative stress and mitochondrial dysfunction act as initiators for neuronal apoptosis events. Peripheral neurotoxicity may be regulated through a decrement in the functioning of the enzymes associated with DNA base excision, the rectification of oxidative impairments, and redox equilibrium. Furthermore, during peripheral neuropathy, apoptosis may arise mediated via augmented p53 activity and the release of cytochrome-C in a mitochondrial pathway [32].

Cisplatin treatment in younger patients often causes *ototoxicity*, with a percent of occurrence above 50%. The symptoms arising in pediatric patients are detrimental and irreversible, and they are represented by bilateral hearing loss, which occurs extremely quickly, tinnitus, and earache. This type of toxicity occurs due to the accumulation of large amounts of cisplatin in cochlear cells, which is helped by the developed activity of copper transporter 1 (Ctr1) and the organic cations transporter 2 (OCT2). In fact, hearing aid cell damage occurs, as previously, due to a high level of oxidative stress caused by the action of cisplatin in forming ROS, as well as a significant reduction in antioxidant protection in the cells [16,33]. A high level of ROS in the cells of the inner ear can trigger cell death via activation of the NADPH oxidase isoform NOX3 that is expressed only in the cochlea, while the death of the outer hair cells of the cochlea can be provoked by cytochrome C releasing and the activating of caspases 9 and 3 [24]. In clinical practice, it has been confirmed that children are significantly more susceptible than adults are to the development of these adverse effects, even if the treatment used lower doses of cisplatin.

Other organ toxicities provoked by cisplatin’s deleterious activity are not expressed to such a significant extent as those mentioned above. Nevertheless, they can cause serious physiological problems and affect the quality of patients’ lives. Cisplatin-induced *hepatotoxicity* has a similar mechanism of development, as in the case of damage to other tissues, and it causes a state of oxidative stress in liver tissue. This type of damage is most often present in patients who receive large doses of this chemotherapeutic agent. Here, oxidative stress can be considered responsible for damage to liver morphology and function, too. Namely, cisplatin, as it is already well known, reduces the level of GSH in liver cells, and this leads to a significant increase in the production of ROS in the mitochondria, an increase in the level of cytokines, and, therefore, an increased risk of cell apoptosis [34,35]. Hepatocyte damage may also be caused by an increased expression of CYP450 [11,36]. Treatment with cisplatin can potentially cause toxicity in cardiac tissue that can be both acute and cumulative, although this is not a very common occurrence. Cisplatin-induced *cardiotoxicity* is characterized by arrhythmias (ventricular arrhythmia, supraventricular tachycardia, atrial fibrillation, sinus bradycardia, etc.); electrocardiography anomalies; thromboembolic; cardiomyopathy; and congestive heart failure [22,30]. The excess in producing high levels of oxidative stress also underlines the arising of cisplatin toxicity in the cardiovascular system [37,38].

### 2.2. Mitochondrial Dysfunction and Inflammatory Responses Induced by Cisplatin

Cisplatin treatment not only results in oxidative stress due to a reduced ability of the body’s endogenous defense to oppose the large amount of ROS that is generated in the process but, on the other hand, imbalances in the functioning of mitochondria occur, as well as the emergence of a pronounced inflammatory response. The mitochondrial dysfunction is a direct consequence of cisplatin accumulation in the mitochondrial matrix, causing redox imbalance. The development of this condition can be registered primarily based on the appearance of histopathological changes such as mitochondria swelling. This is particularly observed in cells that have a high metabolic rate, which implies that they have a very large number of mitochondria. An increased level of ROS is also responsible for changes in the function of respiratory processes in mitochondria, because it affects the reduced activity of mitochondrial respiration complexes I−IV. Even the activity of cytochrome C oxidase can be interrupted by cisplatin. Therefore, the first line of the negative influence of cisplatin represents the detriment of mitochondrial respiration, which leads to an additional increase in ROS. Cisplatin also affects the reduction of calcium absorption in these organelles and can thus promote cell death. This inorganic complex can also affect the reduction of energy production in mitochondria by reducing the peroxisome proliferator-activated receptor (PPAR)-alpha-mediated expression of target genes associated with cellular fatty acid consumption (acyl coenzyme A (acyl-CoA) oxidase and CYP4A1) [29,30,39]. For example, in kidney cells, this cascade of mitochondrial events can even lead to the development of secondary malignancies.

Amador-Martínez et al. [40] explained in detail how the process of mitochondrial dysfunction affects kidney cells and leads to acute kidney injury and, eventually, renal cell apoptosis. After entrance into the cells, cisplatin accumulation in the mitochondria occurs due to the exceptional reactivity of cisplatin with proteins and mitochondrial DNA (mtDNA), while the amount of cisplatin that reacts with nuclear DNA (nDNA) is minimal. The processes underlying cisplatin-induced mitochondrial dysfunction and, thus, acute renal toxicity are closely related to a higher stability of cisplatin–mtDNA reaction products, increasing the concentration of superoxide radical in mitochondria that cannot exit, and mtDNA in comparison to nDNA have a higher mutation rate, which then leads to the development of oxidative stress, inflammation, and apoptosis. High ROS levels induce both structural and functional discrepancies in mitochondria, ultimately causing severe renal tissue injuries [40].

Cisplatin can interact and activate a number of signaling pathways, like MAPK, caspase-3, Bcl-2, p53, and p21, and affect the mitophagy and mitochondrial dynamics, thus causing kidney cell damage [40]. These and many other inflammatory responses can be triggered by cisplatin-induced tissue damage. Inflammation that arises from cisplatin’s negative effects is generally mediated via tumor necrosis factor-alpha (TNF-α) and other cytokines and chemokines. Namely, in kidney cells and other tissues, cisplatin activity leads to this; however, it is also a fact that the picture with other solid tumors is significantly different and that, in those cases, it occurs very often that damage-associated molecular pattern molecules (DAMPs) form as a consequence of tissue damage. These DAMPs are active in the process of releasing chemokines and other cytokines, causing a state of inflammation, in the first line of the activation of toll-like receptor 4 (TLR4) and TNF-α [29,41]. The event of translocation of the transcription factor nuclear factor kappa B (NF-κB) from the cytosol to the nucleus also occurs in this state. The NF-κB pathway induces an increased production and concentration of TNF-α, a proinflammatory cytokine that is generally responsible for the formation of inflammatory processes induced by cisplatin, particularly in kidney cells. Furthermore, TNF-α interacts with endothelial adhesion molecule 1 (ICAM-1), vascular cell adhesion molecule 1 (VCAM-1), and E-selectin to invoke inflammatory cells in tissues. During the inflammation in the renal tubular epithelial cells, TNF-α induces tissue injury and cell death via binding to TNF receptors type 1 and 2 (TNFR1 and TNFR2) [29]. This further leads to the activation of many inflammatory factors and the accumulation of macrophages and neutrophils, which can produce additional quantities of ROS and induce cell toxicity [30]. Another part of the augmentation of inflammation during cisplatin exposure is the activation of the MAPK signaling pathway. This process is highly present, particularly in renal tissues, causing high levels of nephrotoxicity caused by cisplatin-adverse reactions [29,30,42].

## 3. Clinical Implications

Among more than 3000 platinum-based compounds tested as antitumor agents, mainly due to their toxicities, only 7 of them have been approved for individual and/or (more frequent) combinations with other drugs in therapies against different cancer diseases. The synergistic combinations of cisplatin derivatives in the treatment of various malignancies are classified according to the nature of the additional drugs: other anticancer agents (Fluorouracil, Gemcitabine, Cytarabine, Fludarabine, Pemetrexed, Ifosfamide, Irinotecan, Topotecan, Etoposide, Amrubicin, Doxorubicin, Epirubicin, Vinorelbine, Docetaxel, Paclitaxel, and Nab-Paclitaxel); modulators of resistant mechanisms; signaling protein inhibitors (Erlotinib, Bortezomib, and Everolimus); and immunotherapeutic drugs (Atezolizumab, Avelumab, Bevacizumab, Cemiplimab, Cetuximab, Durvalumab, Erlotinib, Imatinib, Necitumumab, Nimotuzumab, Nivolumab, Onartuzumab, Panitumumab, Pembrolizumab, Rilotumumab, Trastuzumab, Tremelimumab, and Sintilimab) [43]. However, there are numerous confirmations that the administration of almost all the mentioned drugs might be accompanied by oxidative damage, therefore making the individual prooxidative effect of those combinations more complex.

### 3.1. Limitations of Cisplatin as a Therapeutic Agent

The toxicity and many side effects of cisplatin are quite well known, because they mostly occur in a large number of patients. Both clinicians and patients are prepared to fight it in order to achieve the most positive outcome of the therapy, which means curing the patient with minimal consequences that cisplatin can cause. However, what represents a cardinal bottleneck in the use of this drug is the occurrence of chemoresistance [44]. The occurrence of cisplatin resistance can be twofold: intrinsic or acquired resistance against cisplatin. In general, intrinsic resistance has not yet been fully investigated, and the mechanisms that cause it have not been fully defined. On the other hand, it has long been known that an acquired resistance is a very common concept and that it mainly arises through two categories of mechanisms. The first group of intracellular events is the one where the drug uptake is reduced, the accumulation is decreased, contrary drug efflux arises, and cisplatin can be inactivated by binding to proteins bearing thiol groups and non-protein molecules with the mentioned group (e.g., glutathione—GSH) or other antioxidants. The second group implies activities related to increased levels of DNA repair, alterations in topoisomerase II, and an increased tolerance to DNA damage [19,24]. There are a very large number of factors that enter into the process of forming a resistance to the action of cisplatin. Many cell cycles and molecules present in the cell are involved in this process. Various phases can be counted there, such as signaling cascades and transcription factors, the repair of DNA lesions, nucleotide excision repair, the mismatch repair pathway (MMR), homologous recombination, replicative bypass, etc. [24].

One of the most comprehensive explanations of the mechanisms of cisplatin resistance was given by Galluzzi et al., who divided them based on the timeline of the development of potential resistance in malignant cells and the functional parameters. Namely, the first changes by occurrence may be when cisplatin is not yet bound to its target molecules in cytoplasm and DNA or pre-target resistance. Thereafter, resistance may be correlated with particular cisplatin-induced damage in the targeted molecules or on-target resistance. Post-target resistance arises in the lethal signaling pathways to which the resulting damage to molecules leads. The last form of cisplatin resistance or off-target resistance may occur in molecules and signaling pathways that are not directly related to the mechanisms of action of cisplatin but may interfere with the process [45].

Decreased intracellular accumulation of cisplatin, a consequence of diminished uptake or augmented efflux, is commonly observed in cell lines with resistance, and enhanced inactivation by intracellular proteins can contribute to that. As already said, cisplatin can undergo covalent binding to GSH, forming a cisplatin–GSH conjugate that prevents the crosslinking of cisplatin and can be eliminated from the cell via the ATP-dependent pump. Modifications in the oncogene expression, like c-fos, c-jun, c-myc, c-abl, and H-ras, and tumor suppressor genes (e.g., p53) have been implicated in cellular resistance to cisplatin as well. Cisplatin-induced damage can be eliminated from the genome by proteins involved in nucleotide excision repair. In addition, the inactivation of mismatch repair genes bestows a resistance to cisplatin. Cell lines with induced cisplatin resistance exhibit substantially elevated levels of repair compared to their parental cell lines, suggesting that DNA repair is one of the crucial phases in the arising of cisplatin resistance. Numerous studies have demonstrated that cisplatin-resistant cells have a greater capacity for adduct tolerance than the corresponding parental cisplatin-sensitive cell lines. Consequently, an enhanced comprehension of the resistance mechanisms operative in vivo can identify potential targets for intervention and potentially augment the applicability of cisplatin in cancer treatment [46].

According to the data so far, cisplatin is the most effective in the treatment of testicular germ cell cancer, with 80% of patients in permanent complete remission. However, it is also a fact that the picture with other solid tumors is significantly different and that, in those cases (ovarian, prostate, lung, and colorectal cancer), cisplatin resistance occurs very often [45].

In addition to the fact that more and more work is being done on this topic in scientific circles, as newly defined model systems are being used in order to determine the way to avoid cisplatin resistance, thus increasing its effectiveness in a larger number of solid tumors, this problem can also be observed as another aspect: namely, how to use some compounds or complex mixtures of active substances that would reduce the unwanted effects of cisplatin and, on the other hand, have no effect or even reduce the characteristics of resistance. Even many natural products have been used for this purpose, to act against tumor drug resistance in different ways. For example, terpenoids andrographolide and pristimerin, as well as flavonoid apigenin, act in various cancer types by inhibiting pro-survival autophagy (inhibits autophagy and promotes cell apoptosis), thus facilitating cisplatin sensitivity [47].

### 3.2. Potential Strategies to Minimize Oxidative Damage

Treatment with cisplatin can trigger the enhanced overproduction of reactive oxygen species within cells, causing a series of chain reactions that leads to deleterious consequences. This is a concentration and time-dependent process, so, with larger amounts and a longer period of exposure, cisplatin manages to overcome the mechanisms of antioxidant protection of the organism and lead to numerous physiological changes and apoptosis as a consequence [16,26]. Thus, oxidative stress should be considered one of the main events that should be taken into account when defining cotreatments to prevent or mitigate the negative effects of cisplatin [25].

Numerous studies have dealt with the application of antioxidant treatments in order to improve cisplatin therapy events. Both natural and synthetic compounds have been used, and many of them have been patented in recent years. For example, the use of some specific lactone compounds are patented as antioxidant agents, among other things, to reduce oxidative stress caused by cisplatin [48]. Recently, the application of some enol compounds (N-(4-acetyl-3,5-dihydroxyphenyl)-2-oxocytclopentane-1-carboxamide (gavinol), 4-N-acetyl-2,6-dihydroxyacetaphenone (NAHA), 2′,4′,6′-trihydroxyacetophenone (THA), and 2-acetylcyclopentanone (2-ACP)) as nucleophilic chemicals in treatment with cisplatin were patented [49]. They are able to lower oxidative stress and, thus, reduce the sensory neuropathy and ototoxicity caused by cisplatin. On the other hand, the same systematic role was observed with nitrated lipids. The use of nitrated fatty acids or esters was patented by Yang [50] to treat organ toxicities induced by chemotherapy, particularly the use of cisplatin.

Besides synthetic antioxidants, various natural compounds with antioxidant action have been studied to lower oxidative stress in the organism exposed to cisplatin. Pure isolated compounds or complex multicomponent mixtures of antioxidants like plant extracts gave great results in the amelioration of cisplatin side effects for which oxidative stress is a characteristic background [51,52]. Some recently patented research results inspire hope that, by applying antioxidants as a mandatory additional therapeutic option in chemotherapy, excellent results may be achieved in accomplishing an equilibrium between deleterious substances and the antioxidative defense, thus protecting the body and increasing the effectiveness of basic therapy. For instance, in 2016, two types of research dealing with the amelioration of cisplatin-induced oxidative stress were patented. The first invention showed that a mixture of *Pineliae Rhizoma* and *Scutellariae Radix* plant extract can serve as significant agents to reduce damage to the gastrointestinal tract and a gastrointestinal motility disorder caused by cisplatin therapy [53]. The second patent dealt with *Elsholtziae Herba* extract as an active ingredient that has been proven can be used for the prevention, improvement, or treatment of acute renal failure, among other things, by reducing the production of ROS [54]. The most recently patented invention represents that the use of brown *Flammulina velutipes* extract can inhibit acute kidney injury caused by cisplatin by lowering the oxidative stress parameters and inflammation response [55].

Nevertheless, the aspect that is rarely emphasized in research studies, although it should be of crucial importance, is the possibility of the interfering of adjuvant therapy with cisplatin, thus diminishing its action and lowering the chemoprotective effects of cisplatin therapy. There have been numerous studies on natural products showing the absence of interaction with cisplatin activity. It was demonstrated that various groups of phytochemicals did not show antagonistic properties toward cisplatin’s main function, e.g., flavonoids, saponins, alkaloids, phenylpropanoids, polysaccharides, napthoquinones, etc. [17]. Some of the natural compounds, such as α-tocopherol (vitamin E), vitamin C, and (−)-epigallocatechin gallate, even showed synergistic effects with cisplatin due to their antineoplastic properties [56,57,58]. The most desirable property of synthetic compounds that could be used as an adjunct to cisplatin therapy is the thiol group protection [59,60].

The largest number of published studies and patents issued for the application of compounds with antioxidant activity in diminishing the negative effects of cisplatin therapy dealt with the application of these compounds in the simplest form, as pure compounds in a solution without any carrier that might improve their action. Nevertheless, the improvements in the fields of compound encapsulation and the synthesis of diverse nanostructures has created an opportunity for antioxidants to be applied and tested in completely new forms with increased efficiency. Nanoparticles have shown great activity in reducing oxidative stress and enhancing cancer treatment [61]. The renal toxicity caused by cisplatin treatment can be successfully treated with polymeric and metallic nanoparticles that mostly act as inhibitors of oxidative stress and scavengers of generated free radicals. This could be found in the overview study by Davoudi et al. [62]. Moreover, lately, synthesized ROS-responsive nanoparticles with “on demand” spatiotemporal release loaded with astaxanthin showed quite promising results in the treatment of oxidative stress during cisplatin-induced ototoxicity [63]. Another novelty in the application of nanoparticles was represented by Zhang and coworkers [64], regarding the implementation of a synergistic antioxidant inhibition nanoplatform to enhance oxidative injury in tumor cells only, thus enabling cisplatin to achieve its antitumor effect without interactions with the antioxidant protection system of cancer cells. This just shows how versatile nanostructures can be used in various aspects of chemotherapy.

Liposomes are the form in which both antioxidants and therapeutic drugs can be encapsulated and distributed. Liposome spheres consist of a hydrophilic core surrounded by a bilayer consisting of phospholipids. They are mostly used in the pharmaceutical industry as delivery systems due to their dual nature; they can encapsulate both hydrophilic and hydrophobic active substances [65]. These vesicles are quite stable and can enhance the bioavailability of encapsulated active compounds [66]. Several studies have shown that this methodology of compound preparation can be effectively used in the treatment of cisplatin side effects [67,68].

## 4. Animal Experimental Models

**Figure 3:**
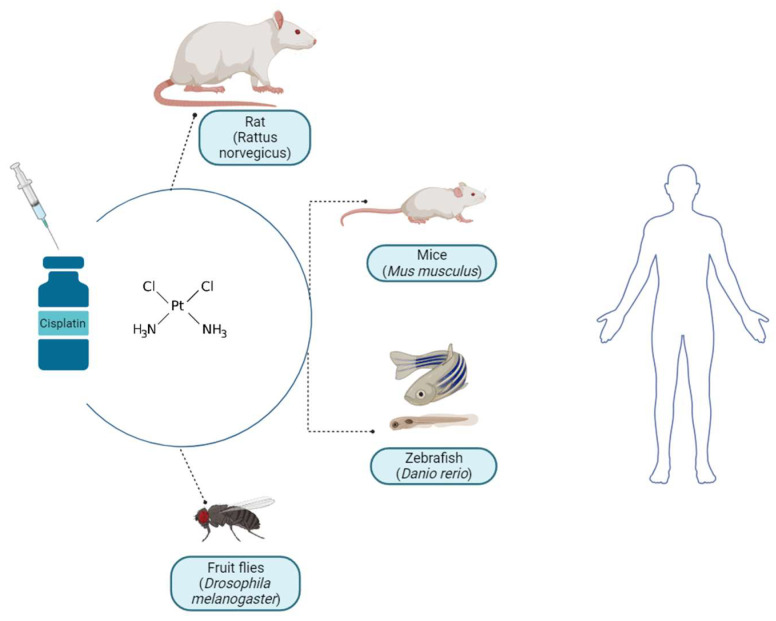
The most frequently used animal models in the in vivo study of cisplatin action. Created in Biorender.com (accessed on 11 September 2023).

Cisplatin is a highly utilized chemotherapeutic agent applied in the treatment of numerous pediatric and adult neoplasms. It is administered in approximately 50% of all anticancer therapies. Therefore, the application of this drug represents a great clinical challenge in terms of the number of side effects it causes. To determine the mechanism of cisplatin activity in the human body, in a therapeutic sense, and when introducing cotreatments that would reduce harmful effects, scientific studies often resort to the use of living subjects who undergo predefined treatments. Most often, it concerns different species of rodents (rats and mice), some knockout species, and, in some cases, dogs are also used, and, in recent times, the use of fruit flies and zebrafish for in vivo tests has become more frequent (Figure 3).

Despite the widespread use of living organisms in cisplatin-related studies, some problems are becoming more common, particularly regarding the use of rodent models. Some previous articles discussed in detail all aspects of cisplatin scientific studies in vivo, the advantages and gaps in treatment design, and the interpretation of the results [69,70]. Generally, cisplatin rodent models are quite simple and exhibit a comparable response to cisplatin intervention as observed in humans. The side effects induced by cisplatin in mice and rats are akin to those experienced by human patients. However, the main messages of more detailed critical observations are as follows. Contrary to the vigilant monitoring and management of cisplatin-associated side effects in cancer patients, animal studies typically narrow their focus on a single aspect of cisplatin toxicity while frequently disregarding or overlooking others. That can be considered one of the most common mistakes. Comprehending the intricacies of cisplatin’s dose- and time-sensitive repercussions and the interconnectivity and interplay between various pathological mechanisms among tissues and organs may enhance the design efficiency of future research and promote a more discerning interpretation of study findings. Acknowledging that not only the absence of comprehensive knowledge and methodologies but also the lack of rigorous and validated animal models for cisplatin are significant factors impeding translatability is crucial [69,70,71]. Another important factor is the increase in cisplatin resistance [24,72].

Considering that many studies have been included in earlier review articles up to 2021, the aim of this article was to review recently published in vivo studies that used cisplatin, to pay attention to the improvements compared to previously established methodologies, and to highlight new ways of investigating cisplatin therapy and side effects.

### Overview of Recent Relevant Animal Models

In order to investigate the effects of cisplatin and various cotreatments in the human population, various animal models are employed. These are mostly mice, rats, and, in some cases, rabbits. Of the greatest importance is the correct design of the experiment, that all activities are ethically fully justified, and that the most comprehensive conclusions can be drawn from the study itself. Table 1 lists some of the most prominent recent studies on the effects of cisplatin using animal models.

Mice and rats comprise an estimated 95% of all living models utilized in laboratory settings, with mice being the most frequently employed species for biomedical research purposes. Several factors contribute to their widespread selection as a preferred animal model. These factors include their size (which simplifies housing and maintenance requirements); rapid reproductive cycles and relatively short lifespans; overall gentle and amiable dispositions; and an extensive knowledge base related to their anatomy, genetic properties, biological processes, and physiological attributes, as well as the ability to breed genetically modified mice and those carrying spontaneous mutations. As mammals, mice exhibit a high degree of similarity with human beings in terms of the form, physiology, and functionality of their organ systems. This further enhances their suitability for use in scientific research aimed at understanding human biology and disease mechanisms. The number of types that are in use for the aforementioned research but also in general research studies is versatile. There can be included models of inbred strains, outbred stocks, spontaneous mutants, genetically engineered mice/“knock-in”/“knock-out”, or transgenic models [99]. Generally, the most often used animal model for inducing acute renal injury by cisplatin are Wistar albino rats, Swiss albino mice, BALB/cN mice, and C57BL/6 mice [100,101,102,103,104,105,106,107,108].

In recently reported scientific studies, the following types of mice have been increasingly used as in vivo models: Kunming, C57BL/6 J, ICR, and BALB/cN mice. Therefore, in the study reported by Tong et al. [73], acute kidney injury was induced in male Kunming mice (6–8 weeks old) using the intraperitoneal injection of cisplatin (20 mg/kg dissolved in saline on the second day) along with treatment with different doses of aspirin for five days. The results showed that aspirin at certain concentrations is able to protect the body from cisplatin-induced acute kidney injury by acting on reducing the inflammatory response that is a consequence of increased oxidative stress, mitochondrial dysfunction, and apoptosis, conditions to which cisplatin leads. In addition, it was concluded that, in the range of activity of the active component that was examined, activation of the AMP-activated protein kinase (AMPK) signaling pathway and peroxisome proliferator-activated receptor-gamma coactivator (PGC-1α) is actually included. Male Kunming mice were also models on which fucoidan-proanthocyanidins nanoparticles were tested to protect in vivo systems against cisplatin-induced acute kidney injury [77]. Animals were also treated with the same dose of cisplatin (20 mg/kg) once on the first day and different concentrations of active component for three days. This research was well combined and supplemented with in vitro tests such as antioxidant and ROS generation assays on HK-2 cells, cell viability, mitochondrial membrane potential, and autophagy. The study reported that the synthesis of nanoparticles of tested compounds can be beneficial for the alleviation of nephrotoxicity caused by cisplatin by the activation of mitophagy and inhibition of the mtDNA-cGAS/STING signaling pathway. Wu et al. [74] tested 7-hydroxycoumarin-β-D-glucuronide in the treatment of nephrotoxicity caused by cisplatin. For that purpose, male C57BL/6 J mice were used and treated with the experiment-tested compound at various concentrations for 3 days, along with cisplatin (10 mg/kg) intraperitoneally (i.p.) on day 3. In this experiment, a twice-lower dose of cisplatin was used, which also caused acute renal failure. Therefore, when designing the previous experimental models, it should be considered whether was it necessary for the concentration of cisplatin to be so high. In this case, it was established that the tested compound alleviated the occurrence of nephrotoxicity by acting on the inhibition of p38 MAPK-mediated apoptosis in living organisms [74]. In addition, male BALB/cN mice have recently been used as model organisms for testing acute renal injury caused by cisplatin [76].

Alkaloid sinomenine was tested to define its protective properties. Toxicity was induced on the second day by the intraperitoneal injection of CP (13 mg/kg). It was shown that the active substance was able to exert its protective nature on the organism by interacting with the many signaling pathways connected with oxidative stress, inflammation, and apoptosis. Nowadays, nanoparticles are synthesized and used more often because of more efficient transport to damaged cells and organs, and better and higher efficiency action is expected [109]. Thus, more and more attention is being paid to nanoparticles on the topic related to the treatment of side effects caused by chemotherapeutics, especially cisplatin. Yan et al. [78] recently used ICR mice to design a model system for the analysis of the effects of various doses of chitosan–tripolyphosphate-encapsulated dihydromyricetin nanoparticles on acute kidney injury caused by cisplatin (10 mg/kg, i.p.) treatment. In addition to in vivo experiments, it is significant that in vitro assays related to nanoparticle properties were also introduced to comprehensively demonstrate the mode of action of the tested substances. It was reported that the tested nanoparticles inhibited oxidative stress and proinflammatory cytokines and activated the Nrf2 signaling pathways, thus ameliorating cisplatin’s deleterious effects in the kidneys. Why it is necessary to look at the problem from several angles and accordingly design the entire study, including animal models, can best be seen from the study reported by Wang et al. [75]. Namely, the authors designed a study based on the network pharmacology and molecular docking of arabinogalactan in order to include animal models. Male ICR mice were used, and acute renal injury was caused by cisplatin (20 mg/kg i.p.) on the seventh day of treatment. First, arabinogalactan was introduced orally in different doses once a day for ten days. The reported results demonstrated the necessity of a multi-analysis approach, predicting potential targets, and showed oxidative stress reduction and the amelioration of kidney cell apoptosis caused by cisplatin.

Hepatotoxicity, although present in cisplatin treatment, is generally rarely the main goal of an animal study, and therefore, a smaller number of studies actually focused on the effect of cisplatin treatment on liver cells. One such recent study was reported by Santos et al. [79], where the idea was to evaluate the role of galectin-3 in liver protection against cisplatin damaging effects. Hepatotoxicity was induced in male Wistar rats by cisplatin injection on three consecutive days (10 mg/kg/day, i.p.). Since, in some groups, galectin-3 was inhibited by modified citrus pectin (MCP) treatment, its effect in reducing hepatic toxicity could be clearly observed. It was noted that the inhibition of galectin-3 resulted in increasing oxidative stress, inflammation, and apoptosis during cisplatin treatment. From the number of parameters, it can be concluded that galectin-3 in hepatocytes has a significant role in protection from cisplatin-induced toxicity. Furthermore, animal models, particularly murine models, serve as a valuable tool for establishing xenograft models by inducing the transplantation of human tumor cells. These models involve the engraftment of human tumor cells either subcutaneously or orthotopically into immunodeficient mice, which lack the ability to reject the human cellular components [110]. In the research study by Gao and coworkers [80], a male BALB/c mice model was used for inducing the xenograft tumor of hepatocellular carcinoma. The in vivo tumorigenesis was generated by the dorsal subcutaneous injection of H22 cells. Treatment of the animals was continued after seven days by injection of aucubin (5 and 10 mg/kg i.p.) or/and cisplatin (5 mg/kg, i.p.) once daily for 1 week. The results revealed that aucubin showed antitumor effects and improved the antitumor efficiency of cisplatin through the suppression of the Akt/β-catenin/PD-L1 axis. Based on this, the conclusion can be reached that such xenograft tumor models are quite advantageous, because it is actually possible to define the complete range of effects of cisplatin on an organism with an already-developed tumor. In addition, they are useful for examining cotreatments not only on the undesired effects of chemotherapy but also on the chemotherapeutic activity of cisplatin itself.

The neurotoxicity of cisplatin, particularly peripheral neuropathy, represents one of the major repercussions of cisplatin treatment [25]. Different models have been developed in order to best induce nerve damage by cisplatin treatment in animals [111,112,113,114]. In addition, various animal models can be used to assess anxiety and depressive behavior, which can also occur as a consequence of cisplatin treatment [18,115,116]. The most recent studies generally used rat models for cisplatin-induced neurotoxicity, probably due to the simpler and easier monitoring and analysis of the changes that occurred, both by experimenters and by software programs. For example, albino Wistar rats were used for the analysis of the neuroprotective activity of lansoprazole against neuronal cell damage caused by cisplatin [81]. Brain toxicity was induced by one dose of cisplatin (10 mg/kg, i.p.) after 5 days of treatment with Lansoprazole (50 mg/kg, p.o.). After various tests for evaluation of the behavioral parameters, like open field and forced swimming tests, many parameters were assessed ex vivo, including primary oxidative stress parameters but also inflammatory parameters and mechanistic studies. The results suggested that the tested compound was able to diminish cisplatin-induced cortical toxicity via multiple signaling pathways, lowering the behavioral changes, inflammation response, and levels of oxidative stress. Another study conducted on Wistar albino rats tested the effects of nanoemulsion formed with *Hypericum perforatum* L. extract on cisplatin-induced cognitive impairment, a state often caused by most chemotherapeutics [82]. After the administration of *Hypericum* nanoemulsion for 21 days, the toxicity in animals was induced by a single dose of cisplatin (10 mg/kg, i.p.). The results showed that the tested material was able to alleviate the negative effects in animals, particularly to lower neurobehavioral alterations and reduce the oxidative stress parameters in brain tissue. The parameters of neuroinflammation were also decreased, along with reduced apoptosis. On the other hand, Sprague–Dawley rats were chosen by Daral et al. [83] as a model system for the analysis of the hippocampus parameters in the state of cisplatin-induced cognitive impairment. The tested substance hypothesized to help reduce the side effects of cisplatin in this experiment was agomelatine administered to the animals daily along with cisplatin (5 mg/kg/week, i.p.) for 4 weeks. The results of a panel of conducted neurobehavioral tests, levels of oxidative stress, and inflammatory parameters, as well as histological observations, showed that the selected treatment was able to improve spatial learning and exploration behaviors. It also reduced neuroinflammation and oxidative stress in the hippocampus of treated animals, standing out as a good candidate for cotreatment of the side effects of cisplatin on the neurological system, potentially interacting with the BDNF/TrkB/nNOS pathways in the hippocampus.

As already mentioned, ototoxicity is one of the most serious problems that can arise primarily in pediatric patients due to cisplatin treatment. In this case, too, animal studies were used to be able to approach the problem from multiple angles and find an adequate solution for the protection of the auditory system from the deleterious effect of cisplatin. One direction is to determine the very mechanism of action of cisplatin. A recent study by Yu et al. [84] dealt with examining how cisplatin damages hearing. Namely, to investigate how Pou4f3 gene mutation can influence pyroptosis in the cochleae of cisplatin-induced deafness, male C57BL/6 mice were used, as well as AAV2-mouse Pou4f3 wild type/mutant, sh-Pou4f3/sh-NC, and sh-NLRP3/sh-NC, for assessing their knockdown and mutation. The dose of cisplatin 20 mg/kg/day (i.p.) administered for 5 consecutive days was enough to induce mice deafness. It was shown that the treatment with cisplatin induced the pyroptosis of cochlear hair cells through the NLRP3/Caspase-3/GSDME pathway and downregulated the Pou4f3 level in the regular mice strain. In the knockout strain of mice, it was clear that this chemotherapy induced auditory damage through the mentioned pathway, especially when the Pou4f3 gene mutation occurred. Another model of ototoxicity was employed by Wang and coworkers [85], who used male C57BL/6 mice for inducing auditory changes with cisplatin (3.0 mg/kg/day, i.p.) for 4 days, followed by 10 days for recovery, for a total of three cycles. Besides in vivo studies, the in vitro treatment of HEI-OC1 cells (an auditory hair cell line) was also introduced. The results showed that ototoxicity was caused by high levels of oxidative stress originating from miR-34a/DRP-1-mediated mitophagy. This was also one of the few studies that mentioned the limitations of the method of examination and the possibilities for improvement. Another example of animal use for models of cisplatin-induced ototoxicity was a study with wild-type C57BL/6 J mice where toxicity was provoked by cisplatin therapy (30 mg/kg, i.p.) [86]. The idea was to inhibit arginine methyltransferase 5 (PRMT5) in order to assess its potential therapy for hearing loss caused by cisplatin. Thus, it was reported based on the obtained results that PRMT5 inhibitors reduced cisplatin-induced hearing loss by acting via the PI3K/Akt-mediated mitochondrial apoptotic pathway. Besides murine models, aquatic models like zebrafish (*Danio rerio*) have been increasingly used recently. Zebrafish have genetic homology with humans, so this model can be used for various purposes to address various toxicities, metabolic syndromes, neurogenerative diseases, inflammation, etc. [117]. The embryos from transgenic zebrafish (Brn3C:EGFP) were recently used for the investigation of the drug esomeprazole’s influence on cisplatin-induced ototoxicity [87]. This type of zebrafish was used because of naturally present neuromasts colored in green, which excluded the need for additional staining and could easily be handled by fluorescence microscopy. Zebrafish embryos were treated with cisplatin (1000 μM) for 5 days post-fertilization and with esomeprazole (2, 20, or 200 μM) for 4 h. As with some of the previous studies, this research was combined with in vitro tests on HEI-OC1 cells. The results indicated that the drug treatment was able to reduce hair cell loss in zebrafish larvae caused by cisplatin, showing an ease and precision when using such an animal model system.

In addition to the development of appropriate animal models for the previously listed, most frequently investigated, adverse effects of cisplatin, models for other injuries that may occur during the use of this chemotherapeutic agent are also in use. For instance, the effects of the plant mixture Wei-Tong-Xin (WTX) on cisplatin-induced changes in gastric antral mucosa were investigated on Kunming mice [88]. Apoptosis in gastric antral mucosa in animals was induced by cisplatin injection (10 mg/kg i.p.), and various concentrations of the tested mixture were given for three consecutive days. With this model, the tested compound was able to reduce the oxidative stress in the gastric antrum of mice and caused the inhibition of the activation of Parkin-dependent mitophagy and apoptosis as negative effects of cisplatin. In combination with in vitro analyses, the overall conclusion was that WTX accomplished its activity towards the elimination of cisplatin side effects on the gastric level. BALB/c mice (6–8 weeks old) were used in a model of colorectal cancer in order to show whether liposomes, with coordinated platinum (II) atoms and a carboxylic group in aspartic acid and glutamic acid, were able to enhance the therapeutic activity of cisplatin [89]. It was shown that liposomes were able to maintain the drug levels in the circulation much longer and had much higher effectiveness against tumor development. The effects of cisplatin on the reproductive system can also be investigated using animal models [35]. A recent model for cisplatin-induced ovarian toxicity was reported in the study by Dinc et al. [90]. The negative effects of cisplatin in the ovaries of albino female Wistar rats were induced by a single daily dose of cisplatin (2.5 mg/kg) for 14 days. A cotreatment with monoterpenoid phenol carvacrol to ameliorate the negative effects of cisplatin was explored. The ovarian tissues were used for analyzing oxidative stress and the inflammatory parameters and histopathological changes. The results showed that higher doses of the tested compound were able to ameliorate ovarian toxicity related to cisplatin by decreasing the inflammation and oxidative stress conditions. Tests on testicular toxicity are mostly done on Wistar rats as well. Nevertheless, we can cite one recent study that used male New Zealand rabbits (8–10 months old) for monitoring testicular damage—to be precise, azoospermia [91]. Generally, it is considered that this is the most frequent breed of rabbits used for research purposes besides European, California, and Dutch-belted rabbits [99]. Thus, in the research conducted by Ismail et al. [91], the rabbits were injected with a single dose of cisplatin (0.7 mL/kg) intratesticular. The tested adipose-derived mesenchymal stem cells (ADMSCs) were administered three days later. The treatment showed significant improvement in the epididymal sperm count, a reduced level of oxidative stress in the testicular tissue, and maintained the hormones at normal physiological levels, in contrast to the cisplatin treatment itself. The zebrafish model is also ideal for the analysis of cisplatin effects on ionocytes, mitochondrion-rich specialized epithelial cells involved in the maintenance of osmotic homeostasis. One such study was published recently by Hung et al. [92], showing the zebrafish as a model for the exploration of cisplatin-induced oxidative stress and apoptosis in ionocytes. Zebrafish (AB strain) embryos were treated with different concentrations of cisplatin solution. They showed that cisplatin reduced labeled ionocytes, the oxidative stress was increased, and apoptosis advanced, followed by the high expression of antioxidative and apoptotic genes. Cisplatin myelosuppression may also be induced in animals, and new methods of suppression of negative effects can be tested. In the study of Xu et al. [93], the authors used mfat-1 transgenic mice in order to analyze the effects of endogenous ω-3 polyunsaturated fatty acids (PUFAs) on cisplatin-induced myelosuppression. In comparison to wild-type mice, it was shown that cisplatin-induced myelosuppression could be prevented by higher levels of ω-3 PUFAs, which act via oxidative damage inhibition and regulation of the NRF2-MDM2-p53 signaling pathway. Fatigue is one of the side effects that can actually be detected in patients without engaging in additional analyses. It represents a big problem during the therapy itself and can continue even after the application of cisplatin is finished [118]. Scott et al. tested male C57BL/6 J mice for the development of cisplatin-induced fatigue and the role of interleukin-10 (IL-10) in the recovery process. The animals were treated with cisplatin for five consecutive days, and the result of decreased voluntary wheel running was shown as a parameter of fatigue-like behavior. By intranasal administration of IL-10 monoclonal antibody IL-10na, the role of IL-10 activity was investigated. Although the results did not confirm this assumption, the model itself performed well in the analysis of cisplatin-induced peripheral neuropathy.

One of the less frequently studied negative activities of cisplatin is cardiotoxicity. Mainly rats are used as an animal model for the purposes of such in vivo experiments. Thus, recent studies, such as the one reported by Yildirim et al. [38], used Sprague–Dawley rats (8–10 weeks old) for evaluating the effects of sinapic acid on cisplatin-induced toxicity. Sinapic acid (20 mg/kg/day) was administered to rats intragastrically for five weeks, while a single dose of cisplatin (3 mg/kg, i.p.) per week was used to induce toxicity in the animals. Based on the monitoring of the biochemical parameters and histological changes in heart tissue, it was concluded that sinapic acid was able to reduce oxidative stress and prevent the development of serious inflammatory conditions, thus mitigating cardiotoxicity in animals. Another recent example of a cardiotoxicity model employed Wistar albino rats to examine a antidiabetic drug used in type 2 diabetes called Canagliflozin (CA) to treat cisplatin-induced changes in the heart structure and function [95]. After 10 days of oral administration of CA (10 mg/kg/day) and toxicity induced by a single dose of cisplatin (7 mg/kg, i.p.) on the 5th day, the serum and heart tissue homogenates were tested on various parameters. The results showed that the use of this drug was helpful in the treatment of cisplatin-induced cardiotoxicity, since the oxidative damage was reduced via the nuclear factor-erythroid 2 related factor 2 (Nrf2) signal and the inflammation was ameliorated via the reduction of the cardiac NO_2_^−^, MPO, iNOS, NF-κB, TNF-alpha, and IL-1β levels, with upregulated levels of the kinases and the p-AKT proteins, Bax, cytochrome C, and Bcl-2 levels.

One of the rarely investigated conditions to which the use of cisplatin can lead is lung disease. Recently, Azithromycin was used to monitor the parameters of oxidative stress in acute lung injury caused by cisplatin [96]. Namely, Azithromycin, a macrolide antibiotic, was given to male albino rats (25 mg/kg/day) for 10 days, and toxicity was caused on day 7 by an intraperitoneal dose of cisplatin (7 mg/kg). The treatment with the tested compound was able to reduce the levels of the oxidative stress parameters like ROS, MDA, NO, MPO, NF-κB p65, TNF-α, and IL-1β and decrease the levels of GSH, SOD, GST, and IL-10. It also mitigated the levels of NF-κB p65 and the proinflammatory parameters. What should definitely be taken into consideration when deciding to test the negative effects of cisplatin (or any other substance) on animal models is, firstly, the justification of the use of living organisms and, then, planning the design of the experiment, where special attention should be focused on “thinking outside the box”, meaning which parameters can be examined during one experiment in order to make the study as efficient as possible and observing the examined problem from several angles. The number of animals necessary for the research must also be taken into account and should be as low as possible. One of these studies was recently published by Mahmoud et al. [97]. They observed the effect of newly synthesized apocynin-chitosan nanoparticles on multiorgan failure (hepatic, cardiac, and renal oxidative injury) caused by cisplatin. Male Wistar albino rats were used as in vivo model organisms and treated with cisplatin (7 mg/kg, i.p.) followed by 5 days of oral application of apocynin-chitosan nanoparticles (135.6 mg/kg). The functional parameters of all the monitored organs were examined, as well as the level of oxidative stress. The results revealed the high amelioration of cisplatin-induced toxicity by apocynin-chitosan nanoparticles through lowering the oxidative stress mediated by Nrf2 activation and reducing the inflammation via NF-κB suppression. Of course, animal models can be used for evaluating the positive effects of cisplatin, too. For example, an animal model was developed for inducing oral ulcerative mucositis with cisplatin and the evaluation of nociception in rats [98]. Male Wistar rats (5–8 weeks old) were pretreated with cisplatin (4 mg/kg/day, i.p.) twice at 4-day intervals, oral ulcerative mucositis was developed using acetic acid, and the defined parameters were assessed. In this scenario, it was reported that cisplatin could be considered for its beneficial effects in reducing inflammation and nociceptive behavior in animals.

By reviewing the listed recent literature, it can be established that the item that is repeated in all tests using animal models is precisely the determination of oxidative stress using different methods of analysis. This indicates that the reduction in oxidative stress and, thus, the prevention of numerous subsequent harmful effects in cisplatin-induced toxicity is the main imperative that every study strives to achieve. Therefore, it is certainly one of the important therapeutic targets for the cotreatment of side effects during chemotherapy that includes cisplatin as the primary chemotherapeutic agent. The level of oxidative stress in serum and in tissue homogenates of animal models can be measured very simply, reliably, and effectively, and therefore, they have widespread research applications.

## 5. Conclusions and Perspectives

Based on the reported data regarding recent improvements in the research of reducing the negative effects of cisplatin, it can be concluded that the current technology is quite advanced, and many new ideas are being applied in order to reach the end result, which is the complete prevention of the manifestation of unwanted effects of these chemotherapeutics. The investigated active compounds were mainly used to alleviate the levels of oxidative stress. The active substances are either obtained from natural sources or synthesized based on molecular design so that they exhibit the appropriate antioxidant effect. Moreover, antioxidants are increasingly applied in new forms, all with the aim of greater bioavailability and targeted action in the desired organ system or corresponding cells. Nanotechnologies have a significant role in the development of suitable therapies for the side effects of cisplatin, and it is believed that this will be the goal that scientific work will strive for in the future.

The fact that the role of animal models in advancing biomedical research remains a crucial aspect of the scientific process must not be ignored. A diverse range of species has been selected based on project-specific objectives and hypotheses, considering factors such as their biological, anatomical, functional, and genetic similarities to humans or other animals. Rodents, particularly mice and rats, dominate the current landscape of experimental subjects in biomedical research, which utilizes approximately 20 million animals annually [117,119]. Despite the numerous discoveries arising from animal experimentation, various limitations and ethical concerns have been raised concerning interspecies differences that may exist in anatomy, metabolism, physiology, and genetics. One primary concern is the inconsistency of the data generated from trials involving animal models. Frequent criticisms include the lack of randomization and blinding and inadequate or no use of formal statistical analysis [119,120]. To address these issues and promote transparency in reporting results from animal studies, Animal Research: Reporting In Vivo Experiments (ARRIVE) guidelines were developed with the information required for scientific publications utilizing animal models. Ethical considerations continue to be an integral part of discussions surrounding the use of animals in research [119].

In conclusion, while animal models will likely remain essential to advancing scientific knowledge in various medical fields, especially for research into the appropriate treatment for cisplatin-induced toxicity, the ongoing ethical dialogue and development of guidelines ensure the continued evolution of the best practices designed to limit unnecessary suffering and enhance the research integrity.

## Figures and Tables

**Table 1:** An insight into some recent representative research dealing with cisplatin therapy and its side effects.

Cisplatin-Induced Damage	Used Animal Model	Treatment	Ref.
Nephrotoxicity (acute kidney injury)	Male Kunming mice (6–8 weeks old)	Aspirin (5/10/20/40 mg/kg dissolved in saline) daily for 5 days + a single intraperitoneal (i.p.) injection of cisplatin (20 mg/kg) on day 2	[73]
Nephrotoxicity (acute kidney injury)	Male C57BL/6 J mice	7-hydroxycoumarin-β-D-glucuronide (7.5, 15, 30 mg/kg) daily for 3 days + cisplatin (10 mg/kg i.p.) on day 3	[74]
Nephrotoxicity (acute kidney injury)	Male ICR mice (8 weeks old)	Network pharmacology analysis + Arabinogalactan (per oral—p.o.—200 and 400 mg/kg, respectively once daily for ten days, 7 days before and 3 days after cisplatin injection) + cisplatin (20 mg/kg i.p.).	[75]
Nephrotoxicity (acute kidney injury)	Male BALB/cN mice (12–14 weeks old)	cisplatin (13 mg/kg, i.p.) + inomenine (5 mg/kg, p.o.) on the third and the fourth day after cisplatin	[76]
Nephrotoxicity (acute kidney injury)	Male Kunming mice	cisplatin (20 mg/kg) once on the first day + Fucoidan-proanthocyanidins nanoparticles (50 and 100 mg/kg, p.o.) once a day for 3 days	[77]
Nephrotoxicity (acute kidney injury)	ICR mice (6 weeks)	CS-DMY-NPs (300, 200, and 100 mg/kg/day) for 5 days + cisplatin (10 mg/kg, i.p.)	[78]
Hepatotoxicity	Male Wistar rats (60–70 days)	MCP on days 1–7 (100 mg/kg/day) + cisplatin on days 8, 9 and 10 (10 mg/kg/day, i.p.)	[79]
Hepatocellular carcinoma (in vivo tumorigenesis)	Male BALB/c mice	a dorsal subcutaneous injection of 2 × 10^6^ H22 cells. At day 7, aucubin (5 and 10 mg/kg i.p.) or/and cisplatin (5 mg/kg i.p.) once daily for 1 week.	[80]
Neurotoxicity	Male albino Wistar rats	Lansoprazole (50 mg/kg, p.o.) + cisplatin (10 mg/kg dose, i.p.) on the 5th day	[81]
Neurotoxicity	Male albino Wistar rats	*Hypericum* nanoemulsion (100 mg/kg) for 21 days + cisplatin (10 mg/kg, i.p.) on day 14	[82]
Neurotoxicity	Male Sprague–Dawley rats (14 weeks)	Agomelatine (40 mg/kg/day, p.o.) and cisplatin (5 mg/kg/week, i.p.) for 4 weeks.	[83]
Ototoxicity	Male C57BL/6 mice (8–12 weeks) and AAV2-mouse Pou4f3 wild type/mutant, sh-Pou4f3/sh-NC and sh-NLRP3/sh-NC	Cisplatin (20 mg/kg/day, i.p.) for 5 consecutive days	[84]
Ototoxicity	Male C57BL/6 mice (6 weeks)	Cisplatin (3.0 mg/kg/day, i.p.) for 4 days, 10 days for recovery (total of three cycles)	[85]
Ototoxicity	Wild-type adult C57BL/6 J mice (7–8 weeks)	Cisplatin (30 mg/kg, i.p.) + PRMT5 inhibitors	[86]
Ototoxicity	Transgenic zebrafish (Brn3C:EGFP) embryos	Cisplatin (1000 μM, 5 days post-fertilization + esomeprazole (2, 20, or 200 μM) for 4 h	[87]
Apoptosis in the gastric antral mucosa	Male Kunming mice	Wei-Tong-Xin (0.5, 1, 2 g/kg, p.o.) for 3 days + cisplatin (10 mg/kg i.p.)	[88]
Colorectal cancer therapy	BALB/c mice (6–8 weeks)	A single bolus tail vein injection of various liposomal formulations and free cisplatin and cisplatin conjugates (3 mg/kg cisplatin equivalent) + 3.5 × 10^5^ C26 cells	[89]
Ovarian toxicity	Albino female Wistar rats	Carvacrol (50 and 100 mg/kg, i.p.) + cisplatin (2.5 mg/kg), all once a day for 14 days	[90]
Testicular damage	Male New Zealand rabbits (8–10 months)	Cisplatin (0.7 mL/kg) injected as a single intra-testicular dose + ADMSCs three days later	[91]
Oxidative stress and apoptosis in mitochondrion-rich ionocytes	Zebrafish (AB strain, 8–12 months) embryos	50, and 100 μM cisplatin solutions (10 embryos in 1 mL of cisplatin solution per well)	[92]
Myelosuppression	mfat-1 transgenic mice, C57BL/6 J mice	7.5 mg/kg cisplatin (once a week for a total of two weeks), a diet containing arachidonic acid	[93]
Fatigue	Male C57BL/6 J mice	cisplatin (2.83 or 2.3 mg/kg/day, i.p.) for five consecutive days	[94]
Cardiotoxicity	Sprague–Dawley rats (8–10 weeks)	Sinapic acid (20 mg/kg/day, intragastrically) for five weeks + a single dose of cisplatin (3 mg/kg/week, i.p.)	[38]
Cardiotoxicity	Wistar albino rats	CA (10 mg/kg/day, p.o.) for 10 days + cisplatin (7 mg/kg, i.p.) on the 5th day	[95]
Acute lung injury	Male albino rats	Azithromycin (25 mg/kg/day) for 10 days + cisplatin (7 mg/kg, i.p.) on day 7	[96]
Multiorgan failure (hepatic, cardiac, and renal oxidative injury)	Male Wistar albino rats	Cisplatin (7 mg/kg, i.p.) Apocynin-chitosan nanoparticles (135.6 mg/kg, p.o.) for 5 days after	[97]
Oral ulcer-induced nociception	Male Wistar rats (5–8 weeks)	Cisplatin (4 mg/kg/day i.p. twice at a 4-day interval)	[98]

## Data Availability

All data are available upon request.
